# Broadening placement opportunities for nursing students through an indirect supervision model

**DOI:** 10.1186/s12912-024-01846-8

**Published:** 2024-07-18

**Authors:** Jonathan Hay, Kate H. Knight, Mark Arnold, Pamela Donaghy-Binks

**Affiliations:** 1 University of Chester, Chester, UK; 2 Liverpool John Moores University, Liverpool, UK; 3 Edge Hill University, Liverpool, UK

**Keywords:** Placement provision, Nursing education, Indirect supervision

## Abstract

**Background:**

Higher Education Institutions struggle to fill national deficits in student placement capacity, especially in social care and the voluntary sector. The Indirect Placement Supervision and Assessment Model and its holistic focus helps redress this deficit.

**Methods:**

A Microsoft Forms survey was distributed to a self-selecting sample of 50 students, placement providers, and university staff, all of whom had been involved in indirect supervision as either an assessor, student, or supervisor.

**Results:**

Three key themes emerged from the data collected; responses suggested that the model generated greater placement variety, increased placement capacity, and brought about reciprocal gains in the learner-supervisor matrix.

**Conclusion:**

The study’s key implication for healthcare institutions pertains to a strong evidence base that the indirect supervision model provides an efficient means of broadening nursing placement variety and capacity alike.

## Introduction

In light of the significant deficit in placement capacity which persists across the UK healthcare landscape at present, it is incumbent on National Health Service England (NHSE) to help redress the shortage and allow Higher Education Institutions (HEIs) to focus upon, and sustainably fund, placement expansion. There is great potential for growth through establishing new placements outside the hospital systems and in non-traditional areas for nursing and allied health (Taylor, et al. 2016, p. 3107) [20]. HEIs offering pre-registration nursing programmes must provide practice learning opportunities for pre-registration learners which meet the criteria required by the Nursing and Midwifery Council (NMC).

As the new NHS Long Term Workforce Plan outlines, the UK government’s ambitious growth targets in relation to nursing practitioners will not support NHS services alone, rather, it will generate a parallel upsurge in primary care and related roles (NHS 2023, p. 131) [6]. Accordingly, it will become increasingly imperative to ensure that pre-registrants have access through their allocated student placements to community, primary and social care providers, in order to “support the medical profession in reshaping itself to meet future patient needs” (NHS 2023, p. 81) [6]. Yet, the implementation of the Standards for Student Supervision and Assessment (SSSA), specifically the requirement that practice assessors and supervisors are health or social care registrants (NMC 2018) [16], is particularly challenging in the context of the social care workforce, due to some areas not having registrants.

HEIs have needed to facilitate novel approaches to student placements in order to meet the NMC requirements; one particular innovation is to fund indirect supervision and assessment (NMC, 2018, Knight, et al. 2022, NMC 2023) [12, 16, 17]. Indirect supervision involves employing practice assessors and supervisors to support the practice placement where health and social care registrants are not available (e.g. charities or social prescribing hubs). Our indirect model is sometimes referred to as a long arm model, the success of which is echoed in the NMC’s expanded guidelines on placement provision (NMC 2023) [17]. This approach has also succeeded in re-establishing students’ ability to undertake innovative placements extending beyond clinical settings, thereby promoting vital alternative skillsets across diverse cultural and situational backgrounds (Knight, et al. 2022) [12]. The para-hospital placements enable the development of a skillset which is increasingly valid in the shifting UK healthcare landscape (Hodge, et al. 2021) [8].

The healthcare needs of deprived communities across the region, alongside driving sustainable healthcare development goals to combat inequalities (CHAMPS, 2021) [2] can be supported by strengthening partnerships between healthcare providers, Private, Independent and Voluntary Organisations (PIVOs)^1^ and Universities in the Cheshire & Merseyside region. From a university level, key to materialising this push is instigating an approach to the student learning experience which actively seeks to foster in pre-registrant practitioners a positive attitude towards addressing the social determinants of health. This step includes an increased focus upon ‘alternative’ community placement opportunities, which allow pre-registration nurses to learn about and address the social inequalities underpinning the UK’s healthcare deficits (Donaghy, et al. 2022a) [4]. This innovative approach aids student recruitment. Recent studies have demonstrated that students have an active desire to learn more about how their skillset can be applied in the contexts of population health and within community care settings (QNI 2022, Donaghy et al. 2022b) [5, 18]. 

The intractability of regulatory frameworks has necessitated that HEIs locate non-standard methods of placement provision, in order to continue to meet pre-registrant uptake of courses (Knight, et al. 2022) [12]. The focus on these types of community placement is in line with the recommendations of the Personalised Care Interprofessional Education Framework (Howarth, et al. 2022) [9]. The findings of this service evaluation also have significance to HEIs in other countries, in establishing the benefits and challenges of adopting indirect student placement or comparable models.

Shifting the placement focus to “the wider determinants of health” is pivotal to addressing healthcare inequalities within the UK healthcare system, since it “helps convey a stronger sense of urgency and importance than focusing on health and wellbeing in general” to those entering the profession (L’Hôte, et al. 2022, p. 8) [13]. Hence, pre-registration students should be made aware from the beginning of their journey as healthcare practitioners that addressing issues centred around the wider determinants of health — whilst focusing upon primary prevention — is vital to their future practice, along with the future of the profession. For vulnerable or underprivileged populations in particular, primary prevention often forms a significant intervention, which can prevent both inpatient and outpatient admissions by addressing their social aetiologies directly, aiding universal health coverage in line with stimulating population health literacy (WHO 2020) [24]. 

Our model offers a significant and ethically sound means of mitigating endemic staffing shortages. Globally, nurses and midwives represent 50% of the current shortfall in healthcare workers — an estimated additional 9 million staff in these professions are needed by 2030 in order to meet demand (WHO 2022) [25]. Although recent data reveals that there was a 4.7 million global staffing uptake in nursing occupations between 2013 and 2018 (WHO 2020), [24] staff shortfall continues to manifest a significant challenge to operational capability in many countries, including the UK. Put simply, “staffing shortages […] are endemic in the UK” healthcare landscape at present (Unruh et al. 2022, p. 434) [21]. Workforce shortages have likewise been exacerbated internationally since the beginning of the COVID-19 pandemic (Chan et al. 2021, Unruh et al. 2022) [3, 21]. This model aims to rebalance the workforce pipeline in both health and social care. Students undertake many health-based placements, but the greatest opportunity for increasing student intake lies in expanding placement circuits. The indirect supervision model achieves these objectives via facilitating supervision within PIVOs which were previously unable to support student placements in line with NMC guidelines (Taylor et al. 2016, Knight et al. 2022).^2^ [20] [11, 12] The objective of this study was to explore the lessons learned to date from a self-selecting sample of students, placement providers, and university staff involved in indirect supervision as an assessor or supervisor.

## Methods

Indirect supervision is an innovative approach to managing practice learning, so a pragmatic approach was taken to service evaluation in the absence of validated tools to formally assess the project (Stake, 2010) [19]. The survey included Likert scale questions to determine participants’ satisfaction and overall experience of the initiative, as well as open ended questions which were aimed at eliciting a qualitative response about service users' experience, an approach which has been used in other placement evaluations (Cant, et al., 2021, Lea et al., 2015) [1, 14]. 

A hyperlink to a Microsoft Forms survey was shared with the students and staff at the two North West Higher Education Institutes who had been involved in an indirect assessed placement. All students involved in the initiative were given equal opportunity to complete the survey. The same link was also shared with the staff in the host organisations. Participation was entirely voluntary, and no financial or other incentive was offered. All participants were asked to confirm that they had read the associated consent form prior to commencing the survey, and informed consent was deemed implicit on submission of the form (Hoyle et al., 2002) [10]. All data collection took place through Microsoft Forms. The study had a sample size of 50.

The data from the survey was aggregated and analysed using thematic analysis, in accordance with the framework proposed by Gale et al. (2013) [7]. Authors analysed the data independently initially, and then compared findings. Themes which had not been identified by multiple participants were discounted as being statistically insignificant. Themes which had been identified by multiple authors were carried forwards. Any discrepancies were discussed until consensus was reached.

## Results

**Fig. 1:**
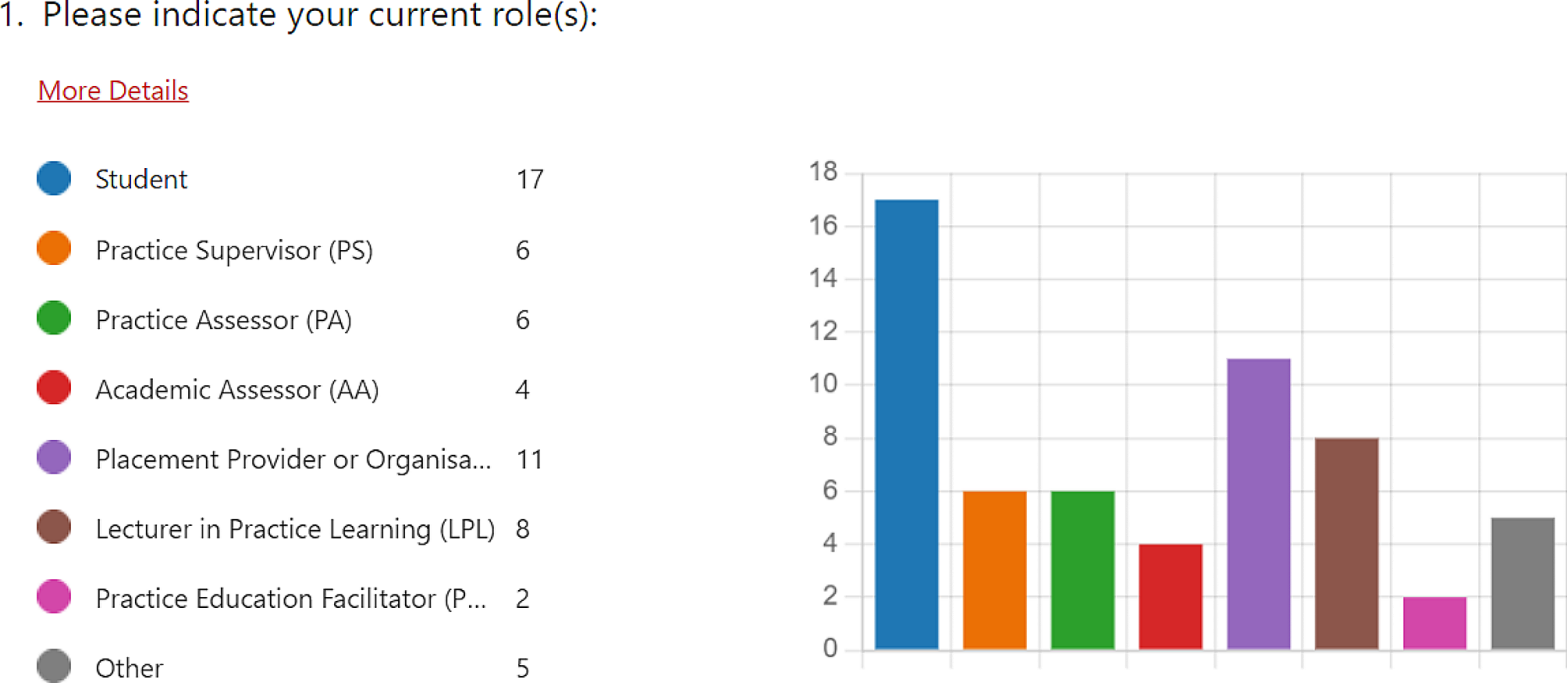
Participant roles

**Fig. 2:**
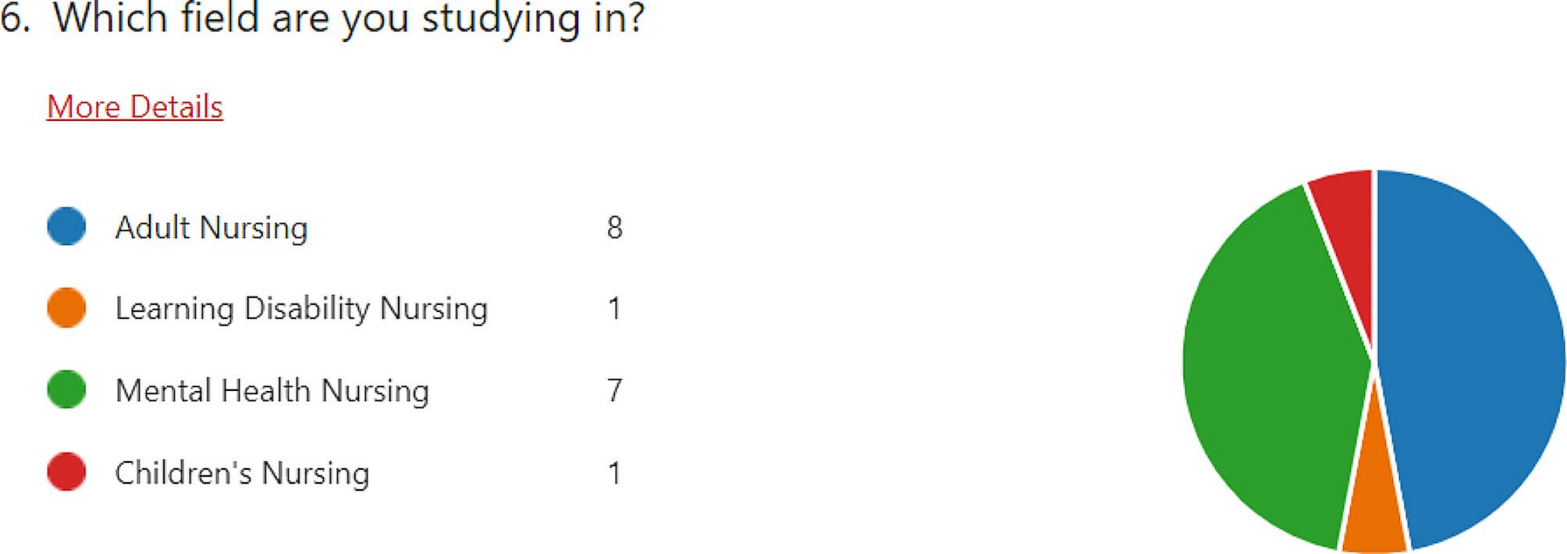
Student participant courses of study

All of the participant data reported in this service evaluation report has been anonymised, and participants have been assigned the acronyms P.1-P.50. Participant names were not collected in any form, and the only identifying data gathered by the researchers concerned participant roles and, where relevant, information concerning which university students were studying at, their course, and level of study. Data from both universities was gathered centrally and is reported here in aggregate, with all references to institution anonymised. In the event that any names are reported in cited feedback, these have been altered to pseudonyms. In total, the survey received 50 responses (*n* = 50), following the subsequent distribution of participant roles (Fig. 1):

The most frequent roles which participants had undertaken within the indirect provision model were students (33% of participants, *n* = 17), Lecturers in Practice Learning (LPLs), and PIVOs (labelled above as Placement Provider or Organisation). Responses which indicated ‘Other’ participant roles included project leads, senior lecturers, cohort leaders, and module leaders. The demographic distribution provides a well-balanced oversight of feedback from participants who have undertaken, or are undertaking, differing roles within the scope of indirect provision. Student participants within the dataset were equally distributed between the participating HEIs, and also equally between levels of study. Nevertheless, as Fig. 2 indicates, there was an uneven distribution of student participants between courses of study:

The results from the service evaluation which are reported below have been coded thematically by the authors, and aspects of the data analysis have also been facilitated by the built-in concordance analysis tools of Microsoft Forms. These inbuilt tools allow for a more objective insight than would otherwise be possible into certain underlying themes in the dataset, such as the determination of key words and themes across participant feedback in aggregate.

When asked to quantitatively rate the overall impact of the indirect model on their practice and/or studies on a five-star scale, the average rating amongst all participants was 4.00*/5.00*. Within this range, 46% of participants (*n* = 23) quantitatively rated the indirect provision model at 5*, with a further 30% of participants (*n* = 15) rating it at 4*. This overall satisfaction level of 76% was also reflected across the wide range of qualitative questions in the survey; responses to these questions are largely reported thematically, with anonymised participant feedback provided where relevant (indicated by italicised text). Minor edits to responses (for matters of spelling, grammar and syntax) have been made on occasion, with care taken to ensure that original meaning is reported accurately.

### Placement Variety

Student feedback on placements included reflections such as: “*I was placed in a community centre in one of England’s most deprived areas. The community centre focused on tackling social determinants of health within the community inclusive of negative mental and physical aspects and addiction*” (P.43), and along similar lines, “*I undertook my […] placement at [redacted], working with patients with drug and alcohol issues. The placement was a great learning experience, and I have gained an insight to the problems which patients with those issues are experiencing*” (P.42), whilst P.48 reported “*I got a realistic view of how hard it is to access mental health support with a dual diagnosis*”. These first-hand experiences suggest that this model has so far provided students with a valuable means of accessing community learning opportunities, and that these experiences have consciously raised their awareness of the increasing validity of these, and similar, soft approaches to healthcare provision. Furthermore, all three of these students quantitatively rated their experience of this model as having been very positive (i.e. the maximum rating).

Despite the overwhelming focus of indirect supervision placements within and around diverse community contexts, much student feedback nevertheless focused on clinical skills acquisition: “*I think this is a brilliant and much needed placement for student nurses. I do suggest that this placement should be given at the end or at the very beginning of the year and to students who have completed all compulsory clinical skills […] as there are no opportunities to practice clinical skills on this placement.*” (P.43). Similarly, another student stated that they appreciated that their indirect placement taught them about “*social prescribing, but it could have been done in a week or a day rather than 6 weeks*” (P.6), epitomising a pervasive and steadfast perspective amongst some that non-traditional placements and forms of experiential learning are less valuable to pre-registrant learners.

Regardless, other students had plainly gained an appreciation for the unique skillsets facilitated by indirect supervision placements: “*I would recommend this [approach] due to being able to have other people experience different environments to progress different skills rather than just clinical skills*” (P.14) And likewise, as another student asserted: “*A nurse must be aware of social determinants of health and look at all aspects of a person’s life. Many factors of a person’s life will influence the physical health of a person. It is important to be aware of bad habits and unfair social determinants and consider the influence of these when diagnosing a person’s illness*” (P.43). These reflections accord far more firmly with both the perspectives of the range of HEI employees involved in assessing students on indirect placements, and the background outlined in this service evaluation. As one PEF related, the approach “*allows students to be placed in a wide variety of organisations, with very experienced and passionate staff, often providing care in community-based organisations, giving an invaluable holistic placement experience.*” (P.34).

This perspective was common amongst other non-student participants. One project lead specified that “*the present levels of deprivation mean many voluntary sector organisations are struggling to meet the needs of communities. Having nursing students with transferable skills and medical knowledge has in some instances contributed positively to the work of local organisations*” (P.15), implying that there are definite workforce benefits from having students attached to indirect placements, not only for PIVOs, but also for their patients and local communities. This assertion aligns very closely with one student reflection that their placement had instilled an “*understanding of the relationship between health and housing and what we can do as nurses to improve the homes our patients return to when they leave our care*” (P.45). Furthermore, an LPL reflected that they had “*enjoyed working as a […] PA building relationships with the students and seeing their progress. Particularly when students were maybe initially disappointed with their placement but then saw the opportunities available to them and were innovative in their achievements*” (P.47).

There was a strong consensus that the indirect supervision model had been “*a huge benefit […] especially in terms of careers and challenging stereotypes*” (P.3), and that, particularly for many students, it had “*made me look at placement areas, opportunities and ways of gaining proficiencies in a different way.*” (P.36). As one particular student noted, it “*taught me about skills that I could use within a hospital/medical setting.*” (P.44), suggesting an active recognition that the soft skills central to indirect supervision placements are not distinct from, but rather, feed into and underpin more traditional routes of clinical practice. Likewise, from the perspective of one PA/PS, it is a “*great model that utilises placements that may not have been used before but are a very valuable learning experience.*” (P.39). These valuable learning experiences can also be extended to the registered practitioners involved in delivery; one AA notes that their involvement “*made me more aware of issues in clinical”* practice-centric approaches to healthcare provision, and highlighted *“the potential variation of practice assessments*” (P.19) which can serve as a means of resolution.

Participants evidenced a strong understanding of the benefits of the civil engagement learning opportunities which indirect placements facilitate. For another student, “*it gave me an opportunity to experience a different environment within the community which had valuable skills and lessons such as working with disabilities and vulnerable people to that capacity.*” (P.14), and for P. 20, “*as an assessor/supervisor I feel it enables students to be placed in more unusual settings and it gives them exposure to settings where clients/patients are looked after that are not in a hospital setting*”. As one student nicely summarises, “*I learnt that there are different places a person can be signposted to after hospital discharge. I learnt of many organisations that are available for people within the community, along with the impact social prescribing can have within a community, and the services outside of hospital and health care settings which are available for people.*” (P.43). Although it was the most prominent, this increased awareness of the value of community engagement amongst practitioners and pre-registrant students alike was far from the only positive theme which emerged from the dataset.

### Increased placement capacity

As one participant emphasised, the indirect supervision model is allowing HEIs to “*open up new and placements in the PIVO sector that have lain dormant [increasing] student capacity within these areas, [and] giving students more insight into the types of nursing posts are available to them when they qualify, giving a further richness to their training.*” (P.50). Other participants also commended the fact that the model “*increases placement capacity*” (P.32), and many also listed adjacent benefits. One participant concluded that the model was a “*great new initiative*” (P.31), and another asserted that it presented a “*more joined up approach.*” (P.46) to student placements than traditional models of assessment, which only offer a partial picture of the contemporary healthcare landscape. Other participants left comments such as “*please do this more often*” (P.12) or “*thanks for letting our organisation be a part of this*” (P.25), which emphasised a depth of gratitude for the model’s implementation.

These highly positive appraisals were often associated with the ability of the model to open new PIVO placements for students, and in turn therefore to expand student cohorts, alongside generating a broader consensus amongst training practitioners of the importance of nursing students learning beyond the NHS. As one participant phrased it, “*the possibilities with this model are endless with opportunities to provide enriching learning experiences, as well as contributing to health promotion initiatives in local neighbourhoods”* (P.15). As one PIVO in particular emphasised, the placements which they had been able to provide through indirect placements would have otherwise been impossible:

*It’s given us an opportunity to have the support of a qualified supervisor for the student and as a result we can provide a student placement. We are learning what’s involved in a model of supervision that we’d not previously experienced. The [indirect supervision] model seems strong on boundaries, we are considering how we can incorporate this element into our own supervisor practice.* (P.26)

Their own institutional practices are currently being reworked in accordance with the example set by the model. This fits in line with statements that the model “*enables better links with university and placement areas*” (P.46), increasing the quality of placement provision in many instances, rather than purely the quantity on hand.

One student reflected that “*I think it’s important to have a placement/experience that is different to your usual placements and can give you something to compare your experiences too. It also allows you to see that there are other possibilities and just how far your nursing can really take you*” (P.49). By repositioning the scope and possibilities of healthcare and nursing provision in this broad sense, the model has the potential to slowly influence student perceptions in a direction that is amenable to population and global health paradigms, with international applicability (van de Mortel 2017) [22]. 

As one PEF concluded, “*many PIVO placements are enthusiastic about placing students, and the opportunity to showcase their organisation, so it allows these areas to place students again*” (P.34), generating a positive feedback loop where successful indirect placements beget subsequent positive indirect placements. In slight contrast, a PS qualified that “*I feel that if a professional relationship and good communication networks are gained from the outset then this model works well. There has to be trust and collaborative working, especially where the student numbers for assessment and supervision are high.*” (P.36). Hence, the gains of the indirect approach are conditional upon a number of factors, at not only the HEI, but also at PIVO and student levels. For one student, “*it can be useful as you have a consistent point of contact […] on placement*” (P.4), and likewise, for a PIVO, the model provided “*good support for both us as the placement provider and the student undertaking the placement”* as a result of a *“knowledgeable and supportive supervisor.*” (P.8). These satisfaction factors are essential to fostering effective and supportive atmospheres throughout placement environments.

### Successes

**Fig. 3:**
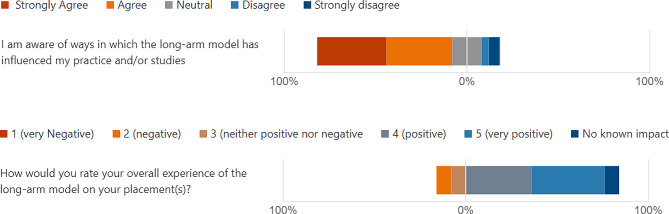
Likert scales illustrating participants’ satisfaction and overall experience

A significant number of further successes were also reported in relation to participants’ experiences of the indirect model. The Likert scales which were used to quantitatively assess participant awareness and overall experience emphasise the dual high levels of satisfaction and engagement with the model (Fig. 3):

The results indicate that a strong majority of participants were both highly aware of the impact of the indirect model on their studies or working pattern, and felt that it had at least aided the provision of an effective placement environment on aggregate. This data evidences that the model has continued to have a strong impact as PIVOs and HEIs transition out of the COVID-19 pandemic, and the unprecedented strain which that period placed upon student placement provision.

As one participant notes, indirect supervision was initially “*implemented due to the sudden shortfall in placements and the need for students to continue to be able to work in practice*” (P.47) throughout the pandemic, whilst strict social distancing measures were in force, but pre-registrant students nevertheless needed to continue gaining competencies and experience with healthcare providers. For one AA, the indirect model proved the perfect means of continuing to train pre-registrant learners: “*the pandemic has affected the qualified staff undertaking assessing courses. That along with sickness means another model would be required. The […] assessor seems to fit this criteria*” (P.16). As one LPL likewise recalls, “*during COVID-19, the model was essential in my role as a health visitor. Without the model students would not have been assessed in practice.*” (P.11).

These early successes of the model have fed into continuing successes, based on participant feedback. For one student:

“*It allowed for me to become integrated into the team and gain deeper awareness into the inner workings of ward-based work. It also allowed for me to gain further experience in the specific field. This allowed for me to gain more confidence and be able to work more independently although still working within my proficiencies. This was evidenced during periods where knowledge of specific individuals was necessary in order to de-escalate situations in which violence was a potentiality.*” (P.23).

Here, the benefits of the model have enabled reciprocal gains; for both the student learner, and for the patients in their care, streamlining the processes necessary to the role. Similarly, participant 33 notes that “*there has been a greater connection between us as LPLs who support students and the PS/PA*” since the model was implemented, drawing the different elements of the new NMC framework together more tightly, and improving team cohesion.

There was certainly a broad consensus that the model is “*Fantastic*” (P.25), even where there were challenges associated in facilitating the placement. For one student who undertook a placement involving a difficult commute:

*It has been time consuming due to the travelling but extremely rewarding to work with the voluntary sector. I have met many inspiring people doing amazing work in local communities and this has been uplifting at a time of crisis. Some organisations have required more initial support than others, but it has been a pleasure to work with them* (P.15).

Other students also made reference to the indirect model having helped them through difficult periods: “*had frequent meetings with the long-arm assessor, this provided me with the support to get through placement*” (P.44); “*my assessor was very professional, understanding, and was a great support during my time on placement.*” (P.42); “*my assessor has been fantastic, she was really supportive, helpful and more than willing to assist with any issues that I may have encountered.*” (P.10). And on the reverse side of the process, for an LPL, “*I feel the implementation has been brilliant. The […] assessors I have linked in with have been fantastic and proactive in the support of learners undertaking these practice learning opportunities*” (P.32).

Overall, feedback on the indirect model was highly positive between staff and students alike, and the model was rated highly regardless of which role each participant had undertaken. A limited amount of suggestions for further improvement in respect of implementation were also evidenced. Qualitative feedback notes that there must be investment in the setting up and preparation of the placements: “*it has been time consuming to chase up which contracts have been signed, I think this process needs streamlining*”, and P.19 voices an analogous suggestion that “*the exchange of information being sent to […] Practice Assessors and those within the organisation should be improved to enhance the experiences of [all parties]. This would reduce the amount of time used to plan meetings, and enable all to be able to attend*”.

## Discussion

**Fig. 4:**
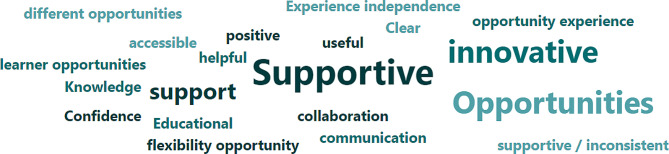
Associative word cloud

The following word cloud resulted from a freeform question asking participants to name the three words they most closely associated with the indirect supervision model (Fig. 4):

There is an overwhelming trend amongst these responses towards positive and active descriptors, reinforcing the data presented earlier that the indirect model is perceived as having an active impact, and that it is perceived to have at least a net positive influence on learning outcomes. Alongside innovation and accessibility, there is likewise a strong emphasis upon the opportunities and new prospects which the model opens up, both for students to practice in new healthcare environments, and for PIVOs to be able to work beyond conventional operational procedures. Even where the word ‘inconsistent’ occurs, it is counterbalanced by an equal number of responses indicating its antithesis, ‘supportive’.

In terms of placement variety, participants responses on aggregate reflected the perspective that indirect supervision was “*brilliant and much needed*” (P.43), as is reflected by the most frequently used descriptive terms in qualitative feedback including the words “opportunity”, “flexibility”, “learner opportunities”, and “accessible”. Contrastingly, for Van Iersel, et al., “there are a number of clear reasons why only few students show an interest for community care”, principally relating to negative preconceptions towards placement variety (Van Iersel, et al., 2018, p. 96) [23]. As that study’s authors conclude, “to recruit student nurses in community care, educational organisations and representatives from the field should collaborate in offering valuable learning experiences that will decrease misconceptions and that foster an optimistic career outlook on this clinical field” (Van Iersel, et al., 2018, p. 96) [23]. The indirect supervision model contributes to healthcare management by providing an educative apparatus which is demonstrably capable of engaging students with a wide variety of community care careers, as is indicated by our results.

Where placement capacity is concerned, our results indicate that the indirect supervision model forms a novel prototype which other HEIs can benefit their placement offerings by implementing. Leon, et al. (2023) [15] recently proposed an alternate model in Australia which likewise proved capable of expanding placement capacity. This model, however, did not fully take into account the diverse needs of community care placements. In contrast, indirect supervision was described by participants as a valuable means of expanding placement capacity, with a focus on community care nursing.

In regard of overall successes, Participant 15 stated an opinion that “*we* […] *need a set of NMC proficiencies which reflect modern nursing practice to value the student learning.*” (P.15). Based on this study’s findings, we contend that the answer is equally to be found in remodelling the current proficiencies via creative new methods of synthesis. As NHS 2023 establishes, it will be essential to bring about a growth in nursing focused on the wider determinants of health, delivered via primary care approaches (NHS 2023, pp. 37, 70) [6]. As a novel model focused on increasing student access to the wider determinants of health, indirect supervision was felt to be effective by a strong majority of participants. When asked whether they would recommend this model of supervision and assessment, 60% of participants (*n* = 30) answered ‘*yes*’, whilst a further 30% (*n* = 15) answered ‘*maybe*’.

## Conclusion

The data gathered indicates that the indirect model is a highly efficient means of providing holistic health and social care-based placements for pre-registrants, and indicates that the model firmly aligns with the future-oriented focus of the new NHS Long Term Workforce Plan. As our results evidence, the indirect model is a highly valuable means of broadening placement opportunities for students, and for reorienting healthcare education towards an emphasis on the wider determinants of health. These results strongly suggest a broad level of approval for the model in its current form, whilst indicating that there remains scope for its implementation to be made increasingly user-friendly. As mentioned above, one area for action that was strongly highlighted by participant feedback is a desire for means to aid advance preparation, and so ease the student journey into the placement environment.

As a next step and an actionable recommendation, we recommend that dissemination of the Indirect Placement Supervision and Assessment Model continues at pace. One potential limitation of this study is that it was conducted on a relatively small cohort, as a result of its purposive sampling approach. The generalisability of our findings will be further tested in follow-up studies. A follow-up study is planned to review students 6–12 months post-graduation, in order to assess the impact of their indirect supervision placements on their practice. This would allow for an understanding of how the model has helped, alongside factors which have inhabited the lessons learned. Such insights would be particularly significant given that there remains little literature to date on graduate practice (van de Mortel, et al. 2017, Donaghy et al. 2022b) [5, 22]..

## Data Availability

Not applicable.

## Footnotes

1 This category encompasses organisations other than direct healthcare providers, such as social care providers and community organisations.

2 For a further overview of the indirect supervision model and its rationale, see Knight et al. 2021.
